# Association between muscle sympathetic nerve activity and red blood cell distribution width in adults

**DOI:** 10.14814/phy2.70577

**Published:** 2025-09-29

**Authors:** Gavin W. Lambert, Carolina Ika Sari, Nora E. Straznicky, John B. Dixon, Nina Eikelis, Murray D. Esler, Markus P. Schlaich, Elisabeth A. Lambert

**Affiliations:** ^1^ Iverson Health Innovation Research Institute Swinburne University of Technology Hawthorn Australia; ^2^ School of Health Sciences Swinburne University of Technology Hawthorn Australia; ^3^ Baker Heart & Diabetes Institute Melbourne Australia; ^4^ Dobney Hypertension Centre, School of Medicine and Pharmacology, Royal Perth Hospital Unit University of Western Australia Perth Australia

**Keywords:** full blood count, hematology, metabolic syndrome, obesity

## Abstract

Measures obtained from the full blood count have been shown to vary around a set point, are stable over years, and provide an indication of disease risk and mortality. In this study, we examined the association between sympathetic nerve activity and components of the full blood count. We performed a retrospective analysis of data drawn from our clinical database. Subjects were included if available data comprised full blood count and muscle sympathetic nerve activity (MSNA). Data were obtained from 160 individuals, comprising healthy volunteers, subjects who were overweight or with clinical obesity, and patients with high blood pressure. MSNA was correlated to the red cell distribution width (RDW, Spearman's rho 0.49 for MSNA b/min and 0.48 for MSNA b/100hb, *p* < 0.001 for both), but bore no association with any of the other blood variables. Regression analysis indicated that the RDW could be predicted by a combination of MSNA and BMI and to the number of components and diagnosis of the metabolic syndrome, measures of insulin resistance, and markers of inflammation. Our observations may provide insight into the possible mechanisms linking the RDW with mortality.

## 1 INTRODUCTION

Red blood cells (RBCs) carry hemoglobin and are responsible for the delivery of oxygen throughout the body. In humans, mature RBCs lack a nucleus, have a biconcave shape with a diameter of 6–8 μm and a volume of 80–100 fL which, depending on physiological and metabolic demand, can increase to 150 fL with membrane distension (macrocytosis) or decrease to less than 60 fL (microcytosis) with membrane contraction (Salvagno et al., 2015). The variability in RBC volume (anisocytosis) may be estimated from the RBC distribution width (RDW) which is calculated from the standard deviation of RBC volume divided by the mean volume of erythrocytes (MCV) multiplied by 100. The RDW normal range varies depending on the equipment used (Salvagno et al., 2015) but is typically in the order of 11%–15% (May et al., 2019). Variation in the RDW is indicative of dysregulation in erythrocyte production and survival and may occur in response to changes in erythropoietin production or responsiveness, with aging, following exercise or in response to metabolic abnormalities (Salvagno et al., 2015).

While the RDW has mostly been used clinically to differentiate between certain types of anaemias (Evans & Jehle, 1991), some reports have focused on the RDW as a marker of chronic disease development, with studies indicating an association of RDW with, for instance, cardiovascular disease and diabetes (Salvagno et al., 2015), dementia (Weuve et al., 2014), carotid intima‐media thickness (Furer et al., 2015), and with increased risk of all‐cause and cardiovascular‐related mortality (Pilling et al., 2018; Tajuddin et al., 2017). Similarly, data from the Third National Health and Nutrition Examination Survey indicated an association between increased RDW and all‐cause mortality (Perlstein et al., 2009). Interestingly, a recent report noted that routine indices derived from the full blood count fluctuate in value around a set point, with the measures being stable over years and, in healthy individuals, provide an indication of subsequent disease risk (Foy et al., 2025). The mechanisms linking RDW with disease development are not known.

Red blood cell synthesis and survival is controlled by erythropoietin (Jelkmann, 2011). Changes in erythropoietin production occur predominantly in response to alterations in tissue oxygenation (Jelkmann, 2011) and, to preserve salt and water, by the renin–angiotensin system (Dunn et al., 2007). Interestingly, interventions that elicit induction of erythropoietin gene expression such as hypoxia (Eckardt et al., 1989) or acclimatization to altitude (Ryan et al., 2014) are also associated with sympathetic nervous activation (Lundby et al., 2018; Morgan et al., 1995). Additionally, erythropoietin may exert more widespread pleiotropic effects, influencing vascular tone and oxygen delivery, skeletal muscle repair, and metabolism, fat mass accumulation, and inflammation in obesity (Suresh et al., 2019).

Given the association between the RDW and cardiovascular disease, there exists evidence also that erythropoiesis may be modulated by the sympathetic nervous system (Al‐Sharea et al., 2019; Robertson et al., 1994). Previous studies by us and others have clearly demonstrated an important role of the sympathetic nervous system in cardiovascular and metabolic disease development (Grassi et al., 2019; Lambert et al., 1995). Whether there is a link between sympathetic nervous activation and the RDW is not known. In this study, we therefore examined the association between sympathetic nerve activity and RDW. We hypothesized that increased RDW in individuals would be directly related to the degree of sympathetic nervous activity and that the association was linked with common cardiometabolic risk factors including blood pressure, obesity, lipids, or markers of glucose control.

## 2 METHODS

This report presents a retrospective analysis of data drawn from our clinical database of studies investigating the regulation of sympathetic nervous activity (Lambert et al., 2010, 2014; Schlaich et al., 2004; Straznicky et al., 2012, 2014). Data obtained from individuals who were examined during the period January 2001–April 2009 (Group 1) and August 2009–September 2014 (Group 2) forms the basis of this report. Participants comprised healthy volunteers, overweight individuals, or individuals with clinical obesity and subjects with high blood pressure. All participants had been weight stable for the preceding 6 months. Exclusion criteria comprised history of heart, cerebrovascular, renal, liver, or thyroid diseases; use of continuous positive airway pressure treatment; and use of medications that could affect study parameters, including oral hypoglycaemic agents, statins, antihypertensive medications, antidepressants, erythropoietin, bisphosphonate, or hormone replacement therapies. Subjects were included if available data included RDW and muscle sympathetic nerve activity (MSNA). Participants were classified as having metabolic syndrome according to the harmonized criteria (Alberti et al., 2009).

Demographic details including age, gender, and clinical history were obtained from standard measurements and questionnaires. All participants had a physical examination prior to investigation. Participants attended our clinic after an overnight fast. Body weight was measured in light indoor clothes without shoes using a digital scale. Resting supine blood pressure was measured 3 times after 5 min of rest using a Dinamap monitor (Model 1846SX, Critikon Inc., Tampa, Florida, USA), and values were averaged. Resting blood samples were obtained from a forearm vein for hematology and biochemical screening. Red blood cell characteristics and hemoglobin concentration were determined using an automated hematology analyser [Beckman Coulter LH780 (Analyser 1) and DXH800 (Analyser 2), Brea, California, USA; Model CD4000 (Analyser 3), Abbott Laboratories, Lake Forest, Illinois, USA]. Given the previous documentation of concordance between analysers (Tan et al., 2011) and that the data drawn from these analysers were obtained during a similar study and time window, data from Analyser 1 and 2 were combined (Group 1). Analyser 3 was used for hematological assessment in Group 2. Plasma glucose and lipid profiles were measured on a commercial analytical system (Architect Analyzer, Abbott Laboratories, Illinois, USA). RDW was determined in triplicate from measures of the MCV using electrical impedance.

MSNA was determined using microneurography as previously described (Lambert et al., 2011). Briefly, a tungsten microelectrode (FHC, Bowdoinham, Maine, USA) was inserted directly into the right peroneal nerve just below the fibular head. A subcutaneous reference electrode was positioned 2–3 cm away from the recording site. The nerve signal was amplified, filtered, and integrated. During MSNA recording, blood pressure was measured continuously using the Finometer system (Finapress Medical System BV, Enschede, The Netherlands), and heart rate was determined from a three‐lead electrocardiogram. Blood pressure, electrocardiogram, and MSNA were digitized with a sampling frequency of 1000 Hz (PowerLab recording system, model ML 785/8SP; ADI Instruments, Sydney, NSW, Australia). Resting measurements were recorded over a 15‐min period and averaged. The MSNA was expressed as burst frequency (burst/min) and burst incidence (burst/100 heart beats).

The distribution of data was evaluated using the Shapiro–Wilk test. The characteristics of the study participants are reported as mean ± standard deviation (SD) for normally distributed data or median (interquartile range) for non‐normal data. Differences between groups were evaluated using analysis of variance. Difference in proportions was evaluated using the Chi square test. The relationship of RDW with MSNA and the anthropometric, biochemical, and hemodynamic variables was estimated using the generalized linear model with RDW as a dependent variable. Predictors with *p* values ≤0.1 for the Spearman's rank correlation coefficient test were selected as independent variables. Multicollinearity among independent variables was assessed using variance inflation factor (VIF) analysis and employed to detect and remove multicollinear predictor variables. The influence of outliers on the regression model was estimated using Cook's distance. Age and BMI were included as covariates and sex and analyser as factors by default. All analyses were performed using R studio (R Studio Team (2015). RStudio: Integrated Development for R. RStudio, Inc., Boston, MA) and Jamovi (version 1.6.15, The Jamovi project, retrieved from https://www.jamovi.org). Data were available from the investigators upon reasonable request.

## 3 RESULTS

Data from 160 subjects were available for analysis (Table 1). Overall, the study cohort was 54% female and included 45% lean or overweight individuals and 55% who had BMI > 30 kg/m^2^. There was no difference in age between female and male subjects (*p* = 0.57). A significant proportion of individuals displayed signs of dyslipidaemia, with healthy total cholesterol levels ≤5.5 mmol/L in 115 individuals, LDL ≤3.5 mmol/L in 119 participants, HDL cholesterol ≥1.0 mmol/L in 132 individuals, and triglycerides ≤1.7 mmol/L in 123 subjects. Most of the cohort (*n* = 141) had fasting blood glucose levels below 6 mmol/L.

**TABLE 1:** Participant demographics and clinical profile.

	Group 1	Group 2	*p*	All subjects	Range
*N* (F/M)	80 (28/52)	80 (59/21)	<0.001	160 (87/73)	
Age (years)	25.0 (15.8)	45.0 (17.0)	<0.001	35.0 (27.0)	18–65
Height (m)	1.72 ± 0.10	1.69 ± 0.09	0.129	1.70 ± 0.10	1.43–1.93
Weight (kg)	77.7 ± 17.2	107.0 ± 20.8	<0.001	92.4 ± 24.1	46.4–180.0
BMI (kg/m^2^)	26.0 (5.0)	36.1 (6.7)	<0.001	31.4 (8.1)	18.0–58.9
Waist (cm)	85.3 ± 13.8	112.0 ± 14.9	<0.001	98.5 ± 19.6	60.0–150.0
Hip (cm)	96.6 ± 13.3	127.0 ± 15.0	<0.001	117.0 + 20.0	70.0–171.0
Waist:Hip ratio	0.87 ± 0.07	0.89 ± 0.08	0.163	0.88 + 0.08	0.7–1.1
Systolic blood pressure (mmHg)	126 ± 24	121 ± 16	0.123	124 ± 20	75–182
Diastolic blood pressure (mmHg)	79 ± 12	70 ± 10	<0.001	75 ± 12	41–107
Heart rate (b/min)	71 (14)	64 (14)	<0.001	68 (15)	48–120
Plasma glucose (nmol/L)	4.6 (0.7)	5.1 (0.8)	<0.001	4.9 (0.9)	3.0–16.0
Plasma insulin	13.2 (8.6)	18.0 (10.9)	0.003	16.4 (10.6)	5.4–71.1
HOMA	2.7 (1.7)	4.4 (3.4)	<0.001	3.8 (3.2)	1.1–24.7
Total cholesterol (mmol/L)	4.5 (1.1)	5.2 (1.1)	<0.001	4.9 (1.3)	0.4–7.3
HDL cholesterol	1.3 (0.5)	3.2 (1.0)	<0.001	1.9 (1.9)	0.8–5.1
LDL cholesterol (nmol/L)	2.1 (1.5)	1.3 (0.8)	<0.001	2.1 (1.5)	0.6–5.8
Triglycerides (nmol/L)	1.2 (0.8)	3.2 (1.0)	<0.001	2.2 (2.0)	0.4–5.1
hsCRP	1.9 (3.3)	3.9 (6.6)	<0.001	3.8 (4.0)	0.2–33.5
Hematocrit (L/L, 0.32–0.42)	0.44 (0.05)	0.40 (0.04)	<0.001	0.42 (0.06)	0.33–0.50
Hemoglobin (g/L, 113–159)	147 ± 15	135 ± 11	<0.001	141 ± 14	101–182
Red cell number (×10^12^/L, 3.6–5.3)	4.9 ± 0.5	4.5 ± 0.4	<0.001	4.7 ± 0.5	3.8–6.6
White cell number	6.5 (1.9)	6.2 (2.5)	0.609	6.4 (2.3)	0.4–12.0
Platelets	256 (75)	247 (90)	0.225	251 (82)	135–694
MCV (fL, 80–97)	87 (5)	89 (5)	0.045	88 (6)	59–97
RDW (%, 10.0–14.5)	11.7 (0.9)	13.4 (0.9)	<0.001	12.6 (1.7)	10.7–17.9
MSNA (b/min)	22 (20)	35 (12)	<0.001	31 (18)	4–77
MSNA (b/100 hb)	35 (25)	58 (22)	<0.001	48 (30)	7–94

*Note*: Insulin and HOMA data were available in 110 participants (36 from Group 1 and 74 from Group 2), and hsCRP was determined in 107 participants (78 from Group 1 and 29 from Group 2).

Abbreviations: HDL, high‐density lipoprotein; HOMA, homeostatic model assessment of β‐cell function; hsCRP, high‐sensitivity C‐reactive protein; LDL, low‐density lipoprotein; MCV, mean red cell volume; MSNA, muscle sympathetic nerve activity; RDW, red cell distribution width.

Sixty participants had high blood pressure based on clinic blood pressure greater than or equal to 140 mmHg systolic or 90 mmHg diastolic blood pressure, and 57 were classified as having the metabolic syndrome. There were significant differences in cardiovascular, metabolic, and hematological parameters between the two groups examined, largely due to the differing focus of the studies from which data were extracted, with participants contributing data to Group 2 being older, 74% female, and having a substantially higher BMI and worse metabolic profile (Table 1). The blood pressure between the two groups was not different. The RDW and MSNA burst frequency and burst incidence were higher in Group 2.

**FIGURE 1:**
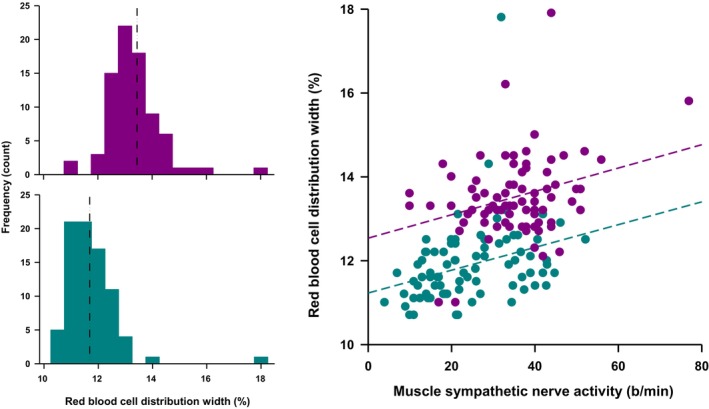
Association between RDW and muscle sympathetic activity. Left panel—Distribution of RDW values among Group 1 (bottom) and Group 2 (top). Right panel—scatter diagram showing association between RDW and MSNA (Group 1 green and Group 2 purple).

**FIGURE 2:**
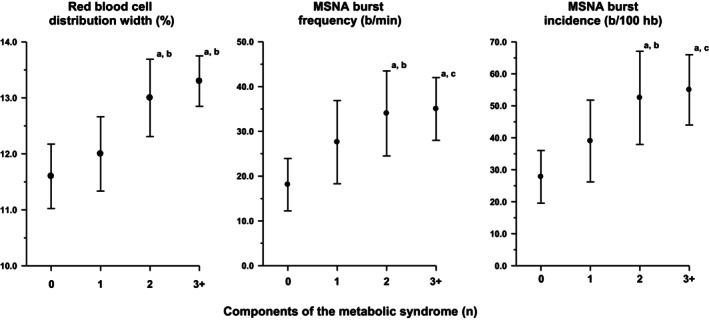
Association between RDW and muscle sympathetic activity and the number of components of the metabolic syndrome. ^a^ *p* < 0.001 compared with 0 components, ^b^ *p* < 0.001 compared with 1 component, and ^c^ *p* < 0.01 compared with 1 component.

Examination of the relationship between MSNA and components of the full blood test indicated that MSNA was correlated with the RDW (Figure 1, Spearman's rho 0.49 for b/min and 0.48 for b/100hb, *p* < 0.001 for both) but bore no association with any of the other blood variables (data not shown). Univariate correlation demonstrated a positive association between the RDW and age and BMI (Spearman's rho 0.35, 0.66 respectively, *p* < 0.001 for both). While RDW values were higher in females [13.1 (1.4%) vs. 12.3 (0.9%), *p* < 0.001], this was likely driven by the increased ratio of females to males in Group 2 where readings were performed using the Abbott Laboratories analyser. Partial correlations controlling for age, sex, and group (analyser) indicated that the RDW was significantly correlated with MSNA, markers of obesity, and glucose control but not with any other factors assessed (Table 2). Similarly, MSNA burst frequency was significantly associated with markers of obesity, plasma insulin, and the HOMA index. The RDW and MSNA were significantly related to the number of components and diagnosis of the metabolic syndrome (Figure 2).

**TABLE 2:** Partial correlations with RDW (Spearman's rho).

	RDW	MSNA (b/min)	MSNA (b/100hb)	Weight	BMI	Waist	eGFR	Glucose	Insulin	HOMA
MSNA (b/min)	0.282 (<0.001)									
MSNA (b/100hb)	0.230 (0.004)	0.934 (<0.001)								
Weight	0.200 (0.013)	0.172 (0.032)	0.142 (0.077)							
BMI	0.257 (0.001)	0.231 (0.004)	0.178 (0.026)	0.892 (<0.001)						
Waist	0.184 (0.021)	0.185 (0.021)	0.143 (0.075)	0.913 (<0.001)	0.876 (<0.001)					
Glucose	0.142 (0.078)	−0.006 (0.942)	−0.042 (0.602)	0.145 (0.073)	0.138 (0.088)	0.203 (0.012)	−0.041 (0.623)			
Insulin	0.231 (0.017)	0.279 (0.004)	0.205 (0.034)	0.141 (0.148)	0.209 (0.031)	0.226 (0.019)	0.061 (0.540)	0.276 (0.004)		
HOMA	0.236 (0.014)	0.264 (0.006)	0.184 (0.058)	0.135 (0.166)	0.190 (0.050)	0.235 (0.015)	0.043 (0.664)	0.435 (<0.001)	0.970 (<0.001)	
hsCRP	0.111 (0.251)	−0.079 (0.416)	−0.161 (0.094)	0.356 (<0.001)	0.425 (<0.001)	0.415 (<0.001)	0.186 (0.057)	0.238 (0.014)	0.137 (0.166)	0.142 (0.154)

*Note*: Partial correlations controlling for age, sex, and group (analyser). Values shown are Spearman's rho (*p*). Only values where *p* was ≤0.1 for RDW, MSNA burst frequency, or burst incidence are shown. Note that insulin and HOMA data were available in 110 participants, and hsCRP was determined in 107 participants.

In the entire cohort, regression analysis indicated that, after controlling for the group (analyser) and sex, the RDW could be predicted by a combination of MSNA and BMI (Table 3). Substitution of weight or waist circumference for BMI, but not waist to hip ratio, revealed similar findings (data not shown). Analysis was repeated in subjects in whom insulin data were available (Table 4). In this abridged group of participants, the RDW could be predicted by a combination of MSNA and insulin (Model 1) or MSNA and HOMA index (Model 2).

**TABLE 3:** RDW multivariate analysis (total cohort).

Independent variables	Estimated coefficient	*F*	*p*	Independent variables	Estimated coefficient	*F*	*p*
Intercept	10.465	22.5	<0.001	Intercept	12.684	20.5	<0.001
Adj *R*‐squared	0.491			Adj *R*‐squared	0.467		
Group	1.013	18.993	<0.001	Group	1.006	17.671	<0.001
Sex	−0.130	0.649	0.422	Sex	−0.147	0.782	0.378
Age	−0.006	1.001	0.319	Age	−0.007	1.051	0.307
MSNA (b/min)	0.027	15.543	<0.001	MSNA (b/100hb)	0.014	8.058	0.005
BMI	0.028	4.473	0.031	BMI	0.032	6.006	0.015
Glucose	0.059	0.860	0.355	Glucose	0.072	1.211	0.273

*Note*: Waist circumference was excluded from analysis due to collinearity with BMI [for MSNA (b/min) analysis, waist circumference VIF = 8.2, for MSNA (b/100hb) analysis, waist circumference VIF = 8.2].

**TABLE 4:** RDW multivariate analysis (abridged cohort).

Independent variables	Estimated coefficient	*F*	*p*	Independent variables	Estimated coefficient	*F*	*p*
*Model 1*							
Intercept	12.853	10.7	<0.001	Intercept	12.835	9.51	<0.001
Adj *R*‐squared	0.416			Adj *R*‐squared	0.384		
Group	0.833	5.438	0.022	Group	0.880	5.745	0.018
Sex	−0.212	0.956	0.331	Sex	−0.264	1.395	0.240
Age	−0.007	0.650	0.422	Age	−0.009	0.902	0.344
MSNA (b/min)	0.031	11.668	<0.001	MSNA (b/100hb)	0.015	5.933	0.017
BMI	0.020	1.366	0.245	BMI	0.023	1.855	0.176
Glucose	0.047	0.380	0.539	Glucose	0.058	0.542	0.463
Insulin	0.020	3.880	0.052	Insulin	0.024	5.282	0.024
*Model 2*							
Intercept	12.860	12.4	<0.001	Intercept	12.842	11.0	<0.001
Adj *R*‐squared	0.422			Adj *R*‐squared	0.390		
Group	0.795	5.086	0.026	Group	0.836	5.305	0.023
Sex	−0.212	0.968	0.327	Sex	−0.265	1.419	0.236
Age	−0.007	0.605	0.439	Age	−0.009	0.844	0.360
MSNA (b/min)	0.032	12.091	<0.001	MSNA (b/100hb)	0.016	6.208	0.014
BMI	0.022	1.858	0.176	BMI	0.027	2.539	0.114
HOMA	0.068	5.006	0.027	HOMA	0.080	6.834	0.010
*Model 3*							
Intercept	12.913	11.1	<0.001	Intercept	12.897	9.87	<0.001
Adj *R*‐squared	0.436			Adj *R*‐squared	0.403		
Group	0.863	6.023	0.016	Group	0.891	6.024	0.016
Sex	−0.125	0.335	0.564	Sex	−0.188	0.704	0.403
Age	−0.005	0.343	0.559	Age	−0.008	0.638	0.426
MSNA (b/min)	0.035	13.935	<0.001	MSNA (b/100hb)	0.019	7.826	0.006
BMI	0.008	0.197	0.658	BMI	0.012	0.439	0.509
HOMA	0.032	0.921	0.340	HOMA	0.045	1.735	0.191
hsCRP	0.054	4.176	0.044	hsCRP	0.056	4.039	0.047

*Note*: Waist circumference was excluded from analysis due to collinearity with BMI. In Models 2 and 3, glucose and insulin were not included in the analysis due to significant collinearity with HOMA index (VIF 8.7 and 19.9, respectively).

The further addition of hsCRP (Model 3) indicated that the RDW could be predicted by a combination of MSNA and hsCRP. Substitution of weight, waist circumference, or waist to hip ratio for BMI revealed similar findings (data not shown).

## 4 DISCUSSION

The full blood count test is commonly used for the diagnosis and monitoring of illness, with changes in parameters being consistent with the diagnosis of a variety of conditions including infection, anemia, certain cancers, and coagulation disorders (Tefferi et al., 2005). While in the acute setting, deviation from the normal range provides important clinical information, a recent study demonstrated that at an individual level, the parameters of the test fluctuate around a stable set point and provide prognostic indication of subsequent disease risk (Foy et al., 2025). In this report, we documented an association between the RDW and sympathetic nervous activity, as assessed from direct measures of MSNA. The relationship between MSNA and the RDW was linked with the presentation of the metabolic syndrome and was associated with the level of adiposity, plasma insulin, the degree of insulin resistance, and the circulating level of hsCRP. The relationship between the RDW and MSNA was significant after controlling for age, sex, and the equipment used for analysis. While our observations may provide insight into the possible mechanisms linking the RDW with mortality, whether sympathetic nervous activation is the primary driver of changes in the RDW or whether the increase occurs secondary to metabolic disturbances associated with weight gain remains unknown.

The generation of red blood cells takes place predominantly within the bone marrow in specific niches. The process is dependent on erythropoietin, the bone marrow microenvironment, and the presence and function of hematopoietic stem and progenitor cells. Recent reports have documented the role that sympathoadrenal activation plays in erythropoietin gene activation by hypoxia (Su et al., 2025) and that brain angiotensin stimulates erythropoiesis via an effect in enhancing sympathetic activity (Rodrigues et al., 2021). While studies have indicated that sympathetic nervous innervation of the stem cell microenvironment is involved in the differentiation, proliferation, and mobilization of hematopoietic stem cells (Fielding & Mendez‐Ferrer, 2020; Katayama et al., 2006; Mendez‐Ferrer et al., 2008), the relationship between erythropoiesis and sympathetic nervous activity is complex.

The observation that erythroid precursor colony growth is reduced in rats following chemical sympathectomy with 6‐hydroxydopamine indicates a role of the sympathetic nervous system in promoting red blood cell generation (Penn et al., 2010). Indeed, at normal physiological levels, noradrenaline exerts a proliferative effect on erythropoiesis (Fonseca et al., 2005; Penn et al., 2010), but at higher concentrations, the growth of pluripotent and erythroid progenitors is inhibited (Fonseca et al., 2005; Penn et al., 2010). Studies by Mendez‐Ferrer et al. (2008) and Golan et al. (2018) have demonstrated that circadian pulses of norepinephrine are required for stem cell differentiation and mobilization via norepinephrine‐induced production of tumor necrosis factor. Peaks in noradrenaline in the morning augment stem cell differentiation and release into the circulation, while melatonin release in the evening supports stem cell retention and renewal (Golan et al., 2018). Whether alterations in these peaks and troughs occur in our clinical and healthy population remains unknown.

Foy and colleagues noted that set point quintiles for RDW and other hematological variables were associated with 10‐year all‐cause mortality and, additionally, that those individuals within the top RDW quintile were more likely to be diagnosed with atrial fibrillation (Foy et al., 2025). While these observations may be consistent with the findings of the present report, given the well‐documented link between obesity and atrial fibrillation (Sha et al., 2024) and that sympathetic nervous activation is evident in patients with atrial fibrillation (Mao et al., 2024), it should be noted that our participants did not present with cardiac rhythm disorders. Single unit MSNA has demonstrated multiple firing of units within a burst in patients with heart rhythm disturbances (Elam & Macefield, 2001; Ikeda et al., 2012). In line with spikes of noradrenaline being associated with hematopoietic stem cell mobilization (Golan et al., 2018), multiple unit firing within a sympathetic burst, in some instances, has been shown to be associated with increased end organ function (Ando et al., 1993) and, at least in the heart, increased noradrenaline release (Lambert et al., 2011). Whether single unit MSNA is associated with the RDW remains unknown.

In contrast to work by Engstrom and colleagues who found that low RDW within the first quartile was associated with the development of diabetes after 14 years of follow‐up (Engstrom et al., 2014), previous studies have shown that the RDW is increased in patients with diabetes (Veeranna et al., 2012), in those with the metabolic syndrome (Sanchez‐Chaparro et al., 2010), and in patients with elevated circulating hsCRP concentration (Lippi et al., 2009). Our analysis provides further support and extends these observations, providing evidence that sympathetic nervous activation associated with weight gain influences RDW either directly via an effect on erythropoiesis or secondarily via an impact on the development of insulin resistance and initiation of inflammation. Alternatively, but not tested in our analysis, disturbances in sleep may contribute to the results obtained. As noted previously, shorter sleep and sleep disturbances, which may occur with, or as a consequence of, weight gain (Figorilli et al., 2025; Kohanmoo et al., 2024), impact negatively on hematopoiesis (McAlpine et al., 2019) and are associated with increased MSNA (Greenlund & Carter, 2022; Tai et al., 2023). The RDW is higher in patients with obstructive sleep apnoea and has been shown to be correlated with the apnoea‐hypopnea index in adults (Sokucu et al., 2012) and children (Morell‐Garcia et al., 2020) but is not reduced following 12 months of continuous positive airways pressure treatment (Leon Subias et al., 2017). Exercise training, which is associated with a reduction in renal noradrenaline spillover (Meredith et al., 1991) and MSNA (Straznicky et al., 2012), results in a diminution in RDW in patients with coronary artery disease (Nishiyama et al., 2016).

Our study is limited by its retrospective and cross‐sectional design and, as such, we do not know the directionality of the MSNA‐RDW relationship and cannot attribute causality. Our data remain hypothesis generating in nature. While our data selection method likely eliminated bias, we cannot exclude that the initial recruitment selected participants who were motivated to engage in health‐related interventions. Whether the findings of the study are representative of the general population is not known. The gender mix in our two groups was skewed due to the nature of the studies from which they were drawn. We attended to this in our statistical analysis, controlling for sex and the analytical equipment used. The clinical characterization of our cohorts and the robust measures of sympathetic activity used are a strength of our study.

In summary, we found an association between the RDW and MSNA. The relationship was associated with metabolic and inflammatory processes associated with obesity. Future prospective studies should be designed to explore the nature of this relationship and to examine whether targeting the sympathetic nervous system or the metabolic consequences of obesity influences RDW and ameliorates the association between RDW, disease development, and mortality.

## FUNDING INFORMATION

No external funding was obtained for the preparation of this manuscript.

## ETHICS STATEMENT

All investigations were approved by the Alfred Hospital Human Ethics Committee, and procedures followed were in accordance with the Helsinki Declaration of 1975, as revised in 2008 and 2024. All subjects gave written informed consent before participating in the various studies.

## References

[phy270577-bib-0001] Alberti, K. G. , Eckel, R. H. , Grundy, S. M. , Zimmet, P. Z. , Cleeman, J. I. , Donato, K. A. , Fruchart, J. C. , James, W. P. , Loria, C. M. , & Smith, S. C. (2009). Harmonizing the metabolic syndrome: A joint interim statement of the International Diabetes Federation Task Force on Epidemiology and Prevention; National Heart, Lung, and Blood Institute; American Heart Association; World Heart Federation; International Atherosclerosis Society; and International Association for the Study of Obesity. Circulation, 120, 1640–1645. 10.1161/CIRCULATIONAHA.109.192644 19805654

[phy270577-bib-0002] Al‐Sharea, A. , Lee, M. K. S. , Whillas, A. , Michell, D. L. , Shihata, W. A. , Nicholls, A. J. , Cooney, O. D. , Kraakman, M. J. , Veiga, C. B. , Jefferis, A. M. , Jackson, K. , Nagareddy, P. R. , Lambert, G. , Wong, C. H. Y. , Andrews, K. L. , Head, G. A. , Chin‐Dusting, J. , & Murphy, A. J. (2019). Chronic sympathetic driven hypertension promotes atherosclerosis by enhancing hematopoiesis. Haematologica, 104, 456–467. 10.3324/haematol.2018.192898 30361420 PMC6395347

[phy270577-bib-0003] Ando, S. , Imaizumi, T. , & Takeshita, A. (1993). Effects of patterns of sympathetic nerve stimulation on vasoconstricting responses in the hindquarter of rabbits. Journal of the Autonomic Nervous System, 45, 225–233. 10.1016/0165-1838(93)90054-x 8106711

[phy270577-bib-0004] Dunn, A. , Lo, V. , & Donnelly, S. (2007). The role of the kidney in blood volume regulation: The kidney as a regulator of the hematocrit. American Journal of the Medical Sciences, 334, 65–71. 17630596 10.1097/MAJ.0b013e318095a4ae

[phy270577-bib-0005] Eckardt, K. U. , Boutellier, U. , Kurtz, A. , Schopen, M. , Koller, E. A. , & Bauer, C. (1989). Rate of erythropoietin formation in humans in response to acute hypobaric hypoxia. Journal of Applied Physiology (1985), 66, 1785–1788. 10.1152/jappl.1989.66.4.1785 2732171

[phy270577-bib-0006] Elam, M. , & Macefield, V. (2001). Multiple firing of single muscle vasoconstrictor neurons during cardiac dysrhythmias in human heart failure. Journal of Applied Physiology (1985), 91, 717–724. 10.1152/jappl.2001.91.2.717 11457786

[phy270577-bib-0007] Engstrom, G. , Smith, J. G. , Persson, M. , Nilsson, P. M. , Melander, O. , & Hedblad, B. (2014). Red cell distribution width, haemoglobin A1c and incidence of diabetes mellitus. Journal of Internal Medicine, 276, 174–183. 10.1111/joim.12188 24471821

[phy270577-bib-0008] Evans, T. C. , & Jehle, D. (1991). The red blood cell distribution width. The Journal of Emergency Medicine, 9(Suppl 1), 71–74. 10.1016/0736-4679(91)90592-4 1955687

[phy270577-bib-0009] Fielding, C. , & Mendez‐Ferrer, S. (2020). Neuronal regulation of bone marrow stem cell niches. F1000Research, 9, 614. 10.12688/f1000research.22554.1 PMC7308883 32595942

[phy270577-bib-0010] Figorilli, M. , Velluzzi, F. , & Redolfi, S. (2025). Obesity and sleep disorders: A bidirectional relationship. Nutrition, Metabolism, and Cardiovascular Diseases, 35, 104014. 10.1016/j.numecd.2025.104014 40180826

[phy270577-bib-0011] Fonseca, R. B. , Mohr, A. M. , Wang, L. , Sifri, Z. C. , Rameshwar, P. , & Livingston, D. H. (2005). The impact of a hypercatecholamine state on erythropoiesis following severe injury and the role of IL‐6. The Journal of Trauma, 59, 884–889. 10.1097/01.ta.0000187653.64300.f5 16374277

[phy270577-bib-0012] Foy, B. H. , Petherbridge, R. , Roth, M. T. , Zhang, C. , De Souza, D. C. , Mow, C. , Patel, H. R. , Patel, C. H. , Ho, S. N. , Lam, E. , Powe, C. E. , Hasserjian, R. P. , Karczewski, K. J. , Tozzo, V. , & Higgins, J. M. (2025). Haematological setpoints are a stable and patient‐specific deep phenotype. Nature, 637, 430–438. 10.1038/s41586-024-08264-5 39663453 PMC12085991

[phy270577-bib-0013] Furer, A. , Finkelstein, A. , Halkin, A. , Revivo, M. , Zuzut, M. , Berliner, S. , Herz, I. , Solodukhin, A. , Ofer, H. , Keren, G. , Banai, S. , & Arbel, Y. (2015). High red blood cell distribution width and preclinical carotid atherosclerosis. Biomarkers, 20, 376–381. 10.3109/1354750X.2015.1096304 26474348

[phy270577-bib-0014] Golan, K. , Kumari, A. , Kollet, O. , Khatib‐Massalha, E. , Subramaniam, M. D. , Ferreira, Z. S. , Avemaria, F. , Rzeszotek, S. , García‐García, A. , Xie, S. , Flores‐Figueroa, E. , Gur‐Cohen, S. , Itkin, T. , Ludin‐Tal, A. , Massalha, H. , Bernshtein, B. , Ciechanowicz, A. K. , Brandis, A. , Mehlman, T. , … Lapidot, T. (2018). Daily onset of light and darkness differentially controls hematopoietic stem cell differentiation and maintenance. Cell Stem Cell, 23, 572–585. 10.1016/j.stem.2018.08.002 30174297

[phy270577-bib-0015] Grassi, G. , Biffi, A. , Seravalle, G. , Trevano, F. Q. , Dell'Oro, R. , Corrao, G. , & Mancia, G. (2019). Sympathetic neural overdrive in the obese and overweight state. Hypertension, 74, 349–358. 10.1161/HYPERTENSIONAHA.119.12885 31203727

[phy270577-bib-0016] Greenlund, I. M. , & Carter, J. R. (2022). Sympathetic neural responses to sleep disorders and insufficiencies. American Journal of Physiology. Heart and Circulatory Physiology, 322, H337–H349. 10.1152/ajpheart.00590.2021 34995163 PMC8836729

[phy270577-bib-0017] Ikeda, T. , Murai, H. , Kaneko, S. , Usui, S. , Kobayashi, D. , Nakano, M. , Ikeda, K. , Takashima, S. I. , Kato, T. , Okajima, M. , Furusho, H. , & Takamura, M. (2012). Augmented single‐unit muscle sympathetic nerve activity in heart failure with chronic atrial fibrillation. The Journal of Physiology, 590, 509–518. 10.1113/jphysiol.2011.223842 22144576 PMC3379697

[phy270577-bib-0018] Jelkmann, W. (2011). Regulation of erythropoietin production. The Journal of Physiology, 589, 1251–1258. 10.1113/jphysiol.2010.195057 21078592 PMC3082088

[phy270577-bib-0019] Katayama, Y. , Battista, M. , Kao, W. M. , Hidalgo, A. , Peired, A. J. , Thomas, S. A. , & Frenette, P. S. (2006). Signals from the sympathetic nervous system regulate hematopoietic stem cell egress from bone marrow. Cell, 124, 407–421. 10.1016/j.cell.2005.10.041 16439213

[phy270577-bib-0020] Kohanmoo, A. , Akhlaghi, M. , Sasani, N. , Nouripour, F. , Lombardo, C. , & Kazemi, A. (2024). Short sleep duration is associated with higher risk of central obesity in adults: A systematic review and meta‐analysis of prospective cohort studies. Obesity Science and Practice, 10, e772. 10.1002/osp4.772 38835720 PMC11149606

[phy270577-bib-0021] Lambert, E. , Sari, C. I. , Dawood, T. , Nguyen, J. , McGrane, M. , Eikelis, N. , Chopra, R. , Wong, C. , Chatzivlastou, K. , Head, G. , Straznicky, N. , Esler, M. , Schlaich, M. , & Lambert, G. (2010). Sympathetic nervous system activity is associated with obesity‐induced subclinical organ damage in young adults. Hypertension, 56, 351–358. 10.1161/HYPERTENSIONAHA.110.155663 20625075

[phy270577-bib-0022] Lambert, E. A. , Rice, T. , Eikelis, N. , Straznicky, N. E. , Lambert, G. W. , Head, G. A. , Hensman, C. , Schlaich, M. P. , & Dixon, J. B. (2014). Sympathetic activity and markers of cardiovascular risk in nondiabetic severely obese patients: The effect of the initial 10% weight loss. American Journal of Hypertension, 27, 1308–1315. 10.1093/ajh/hpu050 24717419

[phy270577-bib-0023] Lambert, E. A. , Schlaich, M. P. , Dawood, T. , Sari, C. , Chopra, R. , Barton, D. A. , Kaye, D. M. , Elam, M. , Esler, M. D. , & Lambert, G. W. (2011). Single‐unit muscle sympathetic nervous activity and its relation to cardiac noradrenaline spillover. The Journal of Physiology, 589, 2597–2605. 10.1113/jphysiol.2011.205351 21486790 PMC3115828

[phy270577-bib-0024] Lambert, G. W. , Kaye, D. M. , Lefkovits, J. , Jennings, G. L. , Turner, A. G. , Cox, H. S. , & Esler, M. D. (1995). Increased central nervous system monoamine neurotransmitter turnover and its association with sympathetic nervous activity in treated heart failure patients. Circulation, 92, 1813–1818. 7545554 10.1161/01.cir.92.7.1813

[phy270577-bib-0025] Leon Subias, E. , de la Cal, S. G. , & Marin Trigo, J. M. (2017). Red cell distribution width in obstructive sleep apnea. Archivos de Bronconeumología, 53, 114–119. 10.1016/j.arbres.2016.05.014 27381970

[phy270577-bib-0026] Lippi, G. , Targher, G. , Montagnana, M. , Salvagno, G. L. , Zoppini, G. , & Guidi, G. C. (2009). Relation between red blood cell distribution width and inflammatory biomarkers in a large cohort of unselected outpatients. Archives of Pathology & Laboratory Medicine, 133, 628–632. 10.5858/133.4.628 19391664

[phy270577-bib-0027] Lundby, C. , Calbet, J. , van Hall, G. , Saltin, B. , & Sander, M. (2018). Sustained sympathetic activity in altitude acclimatizing lowlanders and high‐altitude natives. Scandinavian Journal of Medicine & Science in Sports, 28, 854–861. 10.1111/sms.12976 28948697

[phy270577-bib-0028] Mao, J. , Liu, X. , Kote, A. , Andersson, K. T. , Li, X. , Albert, C. M. , & Chen, P. S. (2024). Skin sympathetic nerve activity in symptomatic and asymptomatic paroxysmal atrial fibrillation. Heart Rhythm, 21, 2437–2444. 10.1016/j.hrthm.2024.06.015 38880203 PMC11608157

[phy270577-bib-0029] May, J. E. , Marques, M. B. , Reddy, V. V. B. , & Gangaraju, R. (2019). Three neglected numbers in the CBC: The RDW, MPV, and NRBC count. Cleveland Clinic Journal of Medicine, 86, 167–172. 10.3949/ccjm.86a.18072 30849034

[phy270577-bib-0030] McAlpine, C. S. , Kiss, M. G. , Rattik, S. , He, S. , Vassalli, A. , Valet, C. , Anzai, A. , Chan, C. T. , Mindur, J. E. , Kahles, F. , Poller, W. C. , Frodermann, V. , Fenn, A. M. , Gregory, A. F. , Halle, L. , Iwamoto, Y. , Hoyer, F. F. , Binder, C. J. , Libby, P. , … Swirski, F. K. (2019). Sleep modulates haematopoiesis and protects against atherosclerosis. Nature, 566, 383–387. 10.1038/s41586-019-0948-2 30760925 PMC6442744

[phy270577-bib-0031] Mendez‐Ferrer, S. , Lucas, D. , Battista, M. , & Frenette, P. S. (2008). Haematopoietic stem cell release is regulated by circadian oscillations. Nature, 452, 442–447. 10.1038/nature06685 18256599

[phy270577-bib-0032] Meredith, I. T. , Friberg, P. , Jennings, G. L. , Dewar, E. M. , Fazio, V. A. , Lambert, G. W. , & Esler, M. D. (1991). Exercise training lowers resting renal but not cardiac sympathetic activity in humans. Hypertension, 18, 575–582. 10.1161/01.hyp.18.5.575 1937659

[phy270577-bib-0033] Morell‐Garcia, D. , Toledo‐Pons, N. , Sanchis, P. , Bauca, J. M. , Sanchez, J. M. , Pena‐Zarza, J. , Giménez, P. , Pierola, J. , de la Peña‐Bravo, M. , Alonso‐Fernández, A. , & Barceló, A. (2020). Red cell distribution width: A new tool for the severity prediction of sleep apnoea syndrome in children. ERJ Open Research, 6, 00278‐2019. 10.1183/23120541.00278-2019 33043053 PMC7533379

[phy270577-bib-0034] Morgan, B. J. , Crabtree, D. C. , Palta, M. , & Skatrud, J. B. (1995). Combined hypoxia and hypercapnia evokes long‐lasting sympathetic activation in humans. Journal of Applied Physiology (1985), 79, 205–213. 10.1152/jappl.1995.79.1.205 7559221

[phy270577-bib-0035] Nishiyama, Y. , Niiyama, H. , Harada, H. , Katou, A. , Yoshida, N. , & Ikeda, H. (2016). Effect of exercise training on red blood cell distribution width as a marker of impaired exercise tolerance in patients with coronary artery disease. International Heart Journal, 57, 553–557. 10.1536/ihj.16-015 27581674

[phy270577-bib-0036] Penn, A. , Mohr, A. M. , Shah, S. G. , Sifri, Z. C. , Kaiser, V. L. , Rameshwar, P. , & Livingston, D. H. (2010). Dose‐response relationship between norepinephrine and erythropoiesis: Evidence for a critical threshold. The Journal of Surgical Research, 163, e85–e90. 10.1016/j.jss.2010.03.051 20605580 PMC2943022

[phy270577-bib-0037] Perlstein, T. S. , Weuve, J. , Pfeffer, M. A. , & Beckman, J. A. (2009). Red blood cell distribution width and mortality risk in a community‐based prospective cohort. Archives of Internal Medicine, 169, 588–594. 10.1001/archinternmed.2009.55 19307522 PMC3387573

[phy270577-bib-0038] Pilling, L. C. , Atkins, J. L. , Kuchel, G. A. , Ferrucci, L. , & Melzer, D. (2018). Red cell distribution width and common disease onsets in 240,477 healthy volunteers followed for up to 9 years. PLoS One, 13, e0203504. 10.1371/journal.pone.0203504 30212481 PMC6136726

[phy270577-bib-0039] Robertson, D. , Krantz, S. B. , & Biaggioni, I. (1994). The anemia of microgravity and recumbency: Role of sympathetic neural control of erythropoietin production. Acta Astronautica, 33, 137–141. 10.1016/0094-5765(94)90118-x 11539513

[phy270577-bib-0040] Rodrigues, A. F. , Todiras, M. , Qadri, F. , Campagnole‐Santos, M. J. , Alenina, N. , & Bader, M. (2021). Increased angiotensin II formation in the brain modulates cardiovascular homeostasis and erythropoiesis. Clinical Science, 135, 1353–1367. 10.1042/CS20210072 34013320

[phy270577-bib-0041] Ryan, B. J. , Wachsmuth, N. B. , Schmidt, W. F. , Byrnes, W. C. , Julian, C. G. , Lovering, A. T. , Subudhi, A. W. , & Roach, R. C. (2014). AltitudeOmics: Rapid hemoglobin mass alterations with early acclimatization to and de‐acclimatization from 5260 m in healthy humans. PLoS One, 9, e108788. 10.1371/journal.pone.0108788 25271637 PMC4182755

[phy270577-bib-0042] Salvagno, G. L. , Sanchis‐Gomar, F. , Picanza, A. , & Lippi, G. (2015). Red blood cell distribution width: A simple parameter with multiple clinical applications. Critical Reviews in Clinical Laboratory Sciences, 52, 86–105. 10.3109/10408363.2014.992064 25535770

[phy270577-bib-0043] Sanchez‐Chaparro, M. A. , Calvo‐Bonacho, E. , Gonzalez‐Quintela, A. , Cabrera, M. , Sainz, J. C. , Fernandez‐Labandera, C. , Aguado, L. Q. , Meseguer, A. F. , Valdivielso, P. , Román‐García, J. , & Ibermutuamur CArdiovascular RIsk Assessment Study Group . (2010). Higher red blood cell distribution width is associated with the metabolic syndrome: Results of the Ibermutuamur CArdiovascular RIsk assessment study. Diabetes Care, 33, e40. 10.2337/dc09-1707 20190288

[phy270577-bib-0044] Schlaich, M. P. , Lambert, E. , Kaye, D. M. , Krozowski, Z. , Campbell, D. J. , Lambert, G. , Hastings, J. , Aggarwal, A. , & Esler, M. D. (2004). Sympathetic augmentation in hypertension: Role of nerve firing, norepinephrine reuptake, and angiotensin neuromodulation. Hypertension, 43, 169–175. 14610101 10.1161/01.HYP.0000103160.35395.9E

[phy270577-bib-0045] Sha, R. , Baines, O. , Hayes, A. , Tompkins, K. , Kalla, M. , Holmes, A. P. , O'Shea, C. , & Pavlovic, D. (2024). Impact of obesity on atrial fibrillation pathogenesis and treatment options. Journal of the American Heart Association, 13, e032277. 10.1161/JAHA.123.032277 38156451 PMC10863823

[phy270577-bib-0046] Sokucu, S. N. , Karasulu, L. , Dalar, L. , Seyhan, E. C. , & Altin, S. (2012). Can red blood cell distribution width predict severity of obstructive sleep apnea syndrome? Journal of Clinical Sleep Medicine, 8, 521–525. 10.5664/jcsm.2146 23066363 PMC3459197

[phy270577-bib-0047] Straznicky, N. E. , Grima, M. T. , Sari, C. I. , Eikelis, N. , Lambert, G. W. , Nestel, P. J. , Karapanagiotidis, S. , Wong, C. , Richards, K. , Marusic, P. , Dixon, J. B. , Schlaich, M. P. , & Lambert, E. A. (2014). A randomized controlled trial of the effects of pioglitazone treatment on sympathetic nervous system activity and cardiovascular function in obese subjects with metabolic syndrome. The Journal of Clinical Endocrinology and Metabolism, 99, E1701–E1707. 10.1210/jc.2014-1976 24937541

[phy270577-bib-0048] Straznicky, N. E. , Lambert, E. A. , Grima, M. T. , Eikelis, N. , Nestel, P. J. , Dawood, T. , Schlaich, M. P. , Masuo, K. , Chopra, R. , Sari, C. I. , Dixon, J. B. , Tilbrook, A. J. , & Lambert, G. W. (2012). The effects of dietary weight loss with or without exercise training on liver enzymes in obese metabolic syndrome subjects. Diabetes, Obesity & Metabolism, 14, 139–148. 10.1111/j.1463-1326.2011.01497.x 21923735

[phy270577-bib-0049] Su, X. , Hildreth, M. , Rapaka, S. , Peng, Y. J. , Nanduri, J. , & Prabhakar, N. R. (2025). Adrenal epinephrine facilitates erythropoietin gene activation by hypoxia through β2 adrenergic receptor interaction with Hif‐2α. American Journal of Physiology—Regulatory, Integrative and Comparative Physiology, 328, R75–R80. 10.1152/ajpregu.00201.2024 39585744 PMC12288984

[phy270577-bib-0050] Suresh, S. , Rajvanshi, P. K. , & Noguchi, C. T. (2019). The many facets of erythropoietin physiologic and metabolic response. Frontiers in Physiology, 10, 1534. 10.3389/fphys.2019.01534 32038269 PMC6984352

[phy270577-bib-0051] Tai, B. W. S. , Dawood, T. , Macefield, V. G. , & Yiallourou, S. R. (2023). The association between sleep duration and muscle sympathetic nerve activity. Clinical Autonomic Research, 33, 647–657. 10.1007/s10286-023-00965-7 37543558 PMC10751264

[phy270577-bib-0052] Tajuddin, S. M. , Nalls, M. A. , Zonderman, A. B. , & Evans, M. K. (2017). Association of red cell distribution width with all‐cause and cardiovascular‐specific mortality in African American and white adults: A prospective cohort study. Journal of Translational Medicine, 15, 208. 10.1186/s12967-017-1313-6 29029617 PMC5640961

[phy270577-bib-0053] Tan, B. T. , Nava, A. J. , & George, T. I. (2011). Evaluation of the Beckman coulter UniCel DxH 800, Beckman coulter LH 780, and Abbott diagnostics cell‐Dyn sapphire hematology analyzers on adult specimens in a tertiary care hospital. American Journal of Clinical Pathology, 135, 939–951. 10.1309/AJCP1V3UXEIQTSLE 21571967

[phy270577-bib-0054] Tefferi, A. , Hanson, C. A. , & Inwards, D. J. (2005). How to interpret and pursue an abnormal complete blood cell count in adults. Mayo Clinic Proceedings, 80, 923–936. 10.4065/80.7.923 16007898 PMC7127472

[phy270577-bib-0055] Veeranna, V. , Zalawadiya, S. K. , Panaich, S. S. , Ramesh, K. , & Afonso, L. (2012). The association of red cell distribution width with glycated hemoglobin among healthy adults without diabetes mellitus. Cardiology, 122, 129–132. 10.1159/000339225 22813786

[phy270577-bib-0056] Weuve, J. , Mendes de Leon, C. F. , Bennett, D. A. , Dong, X. , & Evans, D. A. (2014). The red cell distribution width and anemia in association with prevalent dementia. Alzheimer Disease and Associated Disorders, 28, 99–105. 10.1097/WAD.0b013e318299673c 23751369 PMC3844541

